# Case study report on design, manufacturing and digital representation of a DED-Arc steel node for construction

**DOI:** 10.1038/s41598-026-37315-2

**Published:** 2026-01-23

**Authors:** Johanna Müller, Hendrik Jahns, Marc Müggenburg, Klaus Thiele, Julian Unglaub, Jonas Hensel

**Affiliations:** 1 Chair of Welding Engineering , Chemnitz University of Technology , Reichenhainer Str. 70, 09126 Chemnitz, Germany; 2 Institute of Steel Structures, Technische Universität Braunschweig, Beethovenstraße 51, 38106 Braunschweig, Germany

**Keywords:** Additive manufacturing, Construction, DED-Arc, Slicing, Steel node, Digital twin, WAAM, HSLA, Engineering, Materials science, Mathematics and computing

## Abstract

This report presents a comprehensive investigation into the design, manufacturing, and evaluation of a DED-Arc (also known as Wire Arc Additive Manufacturing, WAAM) Y-Node for the construction industry. While conventional steel nodes for such applications are typically fabricated by welding multiple segments from cut plates or by complex castings, DED-Arc enables individual near-net-shape production of geometrically complex, force-flow optimized components while reducing the need for manual welding and machining. Focusing on the challenges of slicing and manufacturing strategy, such as collision avoidance between the torch and the built component, and guaranteeing torch accessibility in regions with pronounced overhangs, the study highlights the relationship between geometric freedom, path planning complexity, and manufacturing optimization. It emphasizes the importance of early consideration of manufacturing process constraints to enhance design efficiency. The integration of design, manufacturing process, and geometry data within a framework aiming towards a Digital Twin (DT) structure is thoroughly explored with the goal to support a first-time-right fabrication without the need for prototyping, thus reducing material waste. Moreover, the paper demonstrates the role of DT data in predicting component behavior, offering insights into stress distribution predictions influenced by manufacturing strategy. This research contributes to advancing methods for component behaviour analysis and optimization, with significant implications for the construction industry.

## Introduction

The construction industry, a vital sector in the global economy, continually seeks innovative solutions to enhance efficiency, reduce costs, and meet the growing demand for bespoke structures. In this pursuit, automated production processes emerge as promising avenues for achieving more economical construction methods, particularly when increased geometric complexity, functional integration and reduced lead times offset higher process costs^1^. Among these, Additive Manufacturing (AM) stands out as a disruptive technology with the potential to revolutionize the construction landscape over the coming decades^2–6^. AM, a digitalized and automated production method, fabricates components through the successive deposition of materials, primarily employing a layer-by-layer approach, while requiring monitoring and process control to ensure reliable part quality. This technology has a transformative potential to overcome traditional manufacturing hurdles and may enable new possibilities for the construction industry in terms of architectural design freedom.

Structural and architectural designs in construction have historically been shaped by the limitations and capabilities of conventional production methods. AM necessitates a redefinition of design principles to align with the unique characteristics of this innovative manufacturing approach^7^. For structural steel components, the conventional use of semi-finished parts and subsequent processing has been the standard practice.

In the domain of Additive Manufacturing (AM), the Directed Energy Deposition (DED)-Arc process stands out as a technique utilizing gas metal arc welding (GMAW) for the fabrication of metal components^8^. It is particularly suited for the manufacturing of large-scale steel components. The application of steel, especially high-strength steel, holds specific significance for the construction industry. The heightened yield strength inherent in the feed material becomes particularly advantageous, as it allows for a reduction in material consumption. This amplifies productivity, as achieving the same load-bearing capacity requires a diminished quantity of material^9,10^.

One compelling application of the DED-Arc process in construction lies in the manufacturing of force flow-optimized steel nodes. These specialized nodes play a pivotal role as connectors between semi-finished parts, introducing a new dimension of efficiency and structural integrity in construction projects^11–13^. The adoption of DED-Arc for individualized force flow-optimized steel nodes presents a transformative shift from conventional labor-intensive methods used in the fabrication of connection nodes, such as cutting and welding several plate segments or by casting, constrained by geometric limitations. One of the primary advantages lies in the efficiency of the additive manufacturing process. DED-Arc allows for the layer-by-layer deposition of material, enabling the creation of complex geometries with reduced manual effort. This stands in contrast to the traditional approach, which often involves intricate welding and bolting of multiple plates. The additive nature of DED-Arc not only streamlines the manufacturing process but also minimizes the need for extensive manual labor^14^.

While DED offers several advantages, particularly in the production of large-scale components without the need for thermal or mechanical postprocessing of the as-built surface for civil engineering applications^15,16^, a stable process window for different printing strategies needs to be established at first^17,18^. Despite its reputation for offering high geometric freedom, the strategic planning of the printing strategy for force-flow optimized steel components in DED-Arc involves several considerations. A key process-related challenge arises during the slicing of the CAD model^19^, where varying build-up heights in areas with different overhang angles, that also affect mechanical properties^20^, must be addressed. The process intricacies become more pronounced as the degrees of freedom of the manufacturing system come into play. This involves accommodating the dynamic nature of the manufacturing process and ensuring that the printing strategy is able to handle variations in geometry, such as edge defects, layer waviness^21^ or partial layer discontinuities^22^, with special focus on regions with complex overhangs. Furthermore, the system-related challenges extend to limitations imposed by the manufacturing system’s degrees of freedom. The interplay between the design requirements and the manufacturing setup necessitates a thoughtful approach to ensure that the final printed structure meets the desired specifications. This demands a balance between the geometric freedom offered by DED-Arc and the practical constraints dictated by the capabilities and restrictions of the manufacturing system. A critical challenge on the part-level in the planning stage is the avoidance of collisions between the welding torch or robot and the already printed structure. This is particularly crucial in large-scale components, where the risk of interference increases. Integration of collision avoidance strategies into the path planning becomes imperative to ensure a seamless and error-free manufacturing process.

It is noteworthy that these considerations are highly dependent on the specific manufacturing setup, and as of now, they are not fully integrated into the path planning. This underscores the need for ongoing research and development to refine the methodologies for addressing these challenges and further optimizing the DED-Arc process for the efficient and error-free production of large-scale, force-flow optimized steel components in additive manufacturing contexts.

**Fig. 1:**
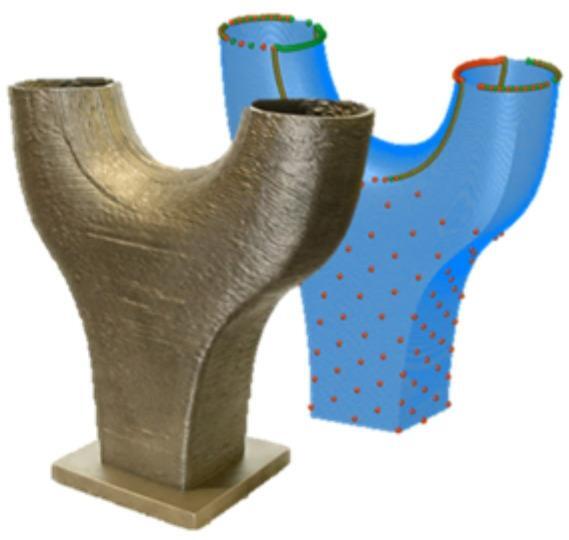
Physical and digital representation of the DED-Arc steel node.

This work offers comprehensive insights into the challenges encountered during the manufacturing and digital representation of a Y-Node, as shown in Fig. 1, which serves as a crucial connector between a rectangular cross-section at the base, seamlessly transitioning into two round profiles. While the geometry itself may be of less relevance in real structures, it was chosen due to its complexity yielding challenges in manufacturing and component testing. The presented work contributes to advancing additive manufacturing in construction by providing a detailed case study that links process strategy, geometric complexity, and predictive assessment of component performance.

## Manufacturing of a Y-Node using DED-Arc

The DED-Arc process holds considerable significance within the construction sector due to its reasonable build-up rate. The process enables the design and manufacturing of unique, individualized components with a notable level of geometric freedom compared to conventional manufacturing techniques. However, it is imperative to acknowledge inherent limitations and manufacturing constraints during the design phase, such as torch accessibility, minimum build angles and maximum overhang angles. The realization of optimal geometric freedom necessitates the implementation of an 8-axis manufacturing system, incorporating a tilt-turn table, respectively a manipulator.

**Fig. 2:**
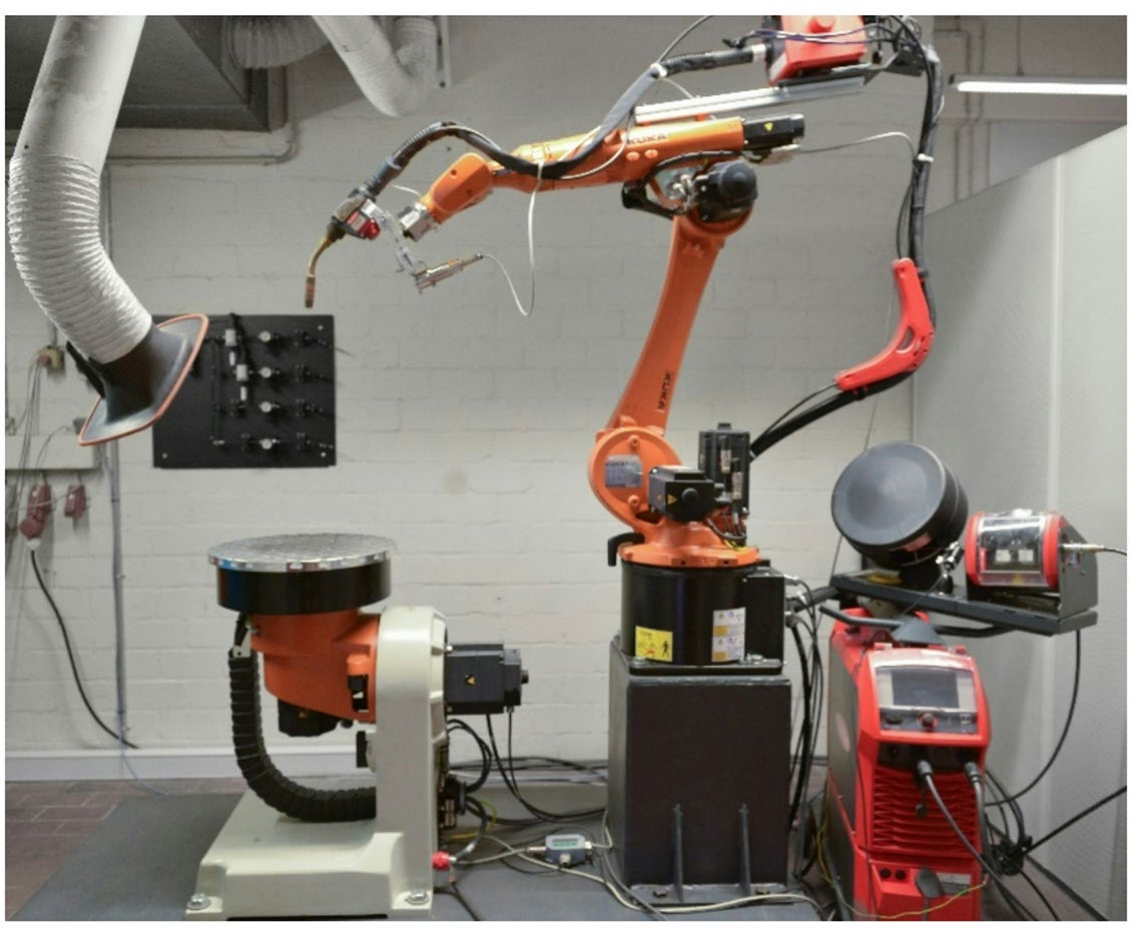
DED-Arc manufacturing system (8-axis).

The specific manufacturing setup employed for fabricating the Y-Node, as illustrated in Fig. 2, involved a 6-axis KUKA robot equipped with a tilt-turn table. Slicing and path planning were executed using the DCAM software developed by S.K.M. Informatik. The deposition material utilized was a high-strength low-alloy (HSLA) steel electrode with a diameter of 1.2 mm, and its chemical composition is given in Table 1. Shielding gas M21 (82% Ar, 18% CO_2_) was used with a flow rate of 14 L/min during manufacturing. The DED-Arc process parameters (current, voltage and wire feed rate) were measured during the manufacturing using a welding process scanner (HKS Weldscanner).

**Table 1:** Measured chemical composition of wire electrode Böhler welding 3Dprint AM 80 HD.

Chemical composition (wt.-%)											
C		Si	Mn	P	S	Cr	Mo	Ni	Al	Cu	Fe
0.11		0.36	1.7	0.01	0.003	0.38	0.6	2.18	0.01	0.055	bal.

As a power source a Fronius TPS500i with the CMT Dynamic characteristic curve was utilized. In the context of DED-Arc processes, a delicate equilibrium must be maintained between achieving a high build-up rate and ensuring substantial geometric freedom. Modifying the energy input, and consequently the build-up rate, influences the molten pool and interlayer cooling times, in turn affecting the geometry, the microstructure and thus the resulting mechanical properties^10,23^. A high energy input proves disadvantageous for printing in overhang positions as it leads to a higher geometric variability of the as-built surface, therefore impacting the mechanical properties of the resultant structure negatively^15,24^. Previous investigations focused on identifying optimal process parameters that yield superior mechanical properties^10^. The resulting low energy input process parameters are comprehensively presented in Table 2.

**Table 2:** DED-Arc process parameters.

Torch travel speed (cm/min)	Wire feed rate (m/min)	Current (A)	Voltage (V)	Energy input (kJ/cm)	Interpass temperature (°C)
25	2.0	77	13	2.4	< 200

### Slicing and path planning

#### Manufacturing strategy of the node

Prior to delving into the intricacies of slicing and path planning for the Y-Node fabrication, a thorough analysis of its geometry becomes imperative to define an effective manufacturing strategy. A critical aspect of such nodes involves the structural configuration of the bridge situated in the central region. This region encompasses the convergence of the main body, subsequently diverging into two branches characterized by a rounded profile.

**Fig. 3:**
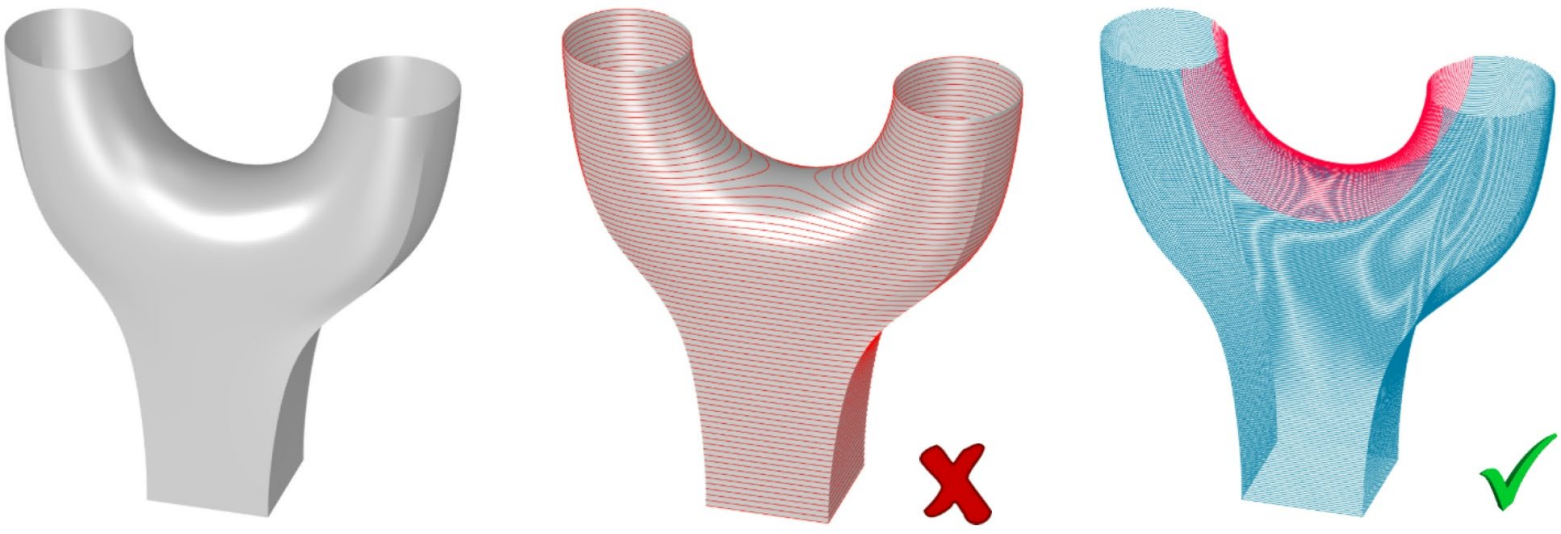
Rendering of the structure (left); Planar slicing (middle); Separation of the node into main body (blue) and bridge structure (red) as manufacturing strategy for the avoidance of unfavorable overhangs, crossing points and sharp radii in the strong overhang areas (right).

Upon considering the entire node during the slicing process, the path for the bridge manifests sharp radii coupled with a pronounced overhang, as depicted in Fig. 3. This configuration poses a considerable risk when employing an electric arc, as the torch orientation necessitates rapid adjustments, and there exists a heightened susceptibility to arc deflection, potentially leading to insufficient fusion. To mitigate these risks, a strategic decision is made to segregate the Y-Node into distinct components: the main body and the bridge. Notably, the build-up direction for the bridge undergoes alteration, and a deliberate midpoint closure is implemented to enhance overall fabrication stability and mitigate potential printing challenges.

#### Slicing methods

**Fig. 4:**
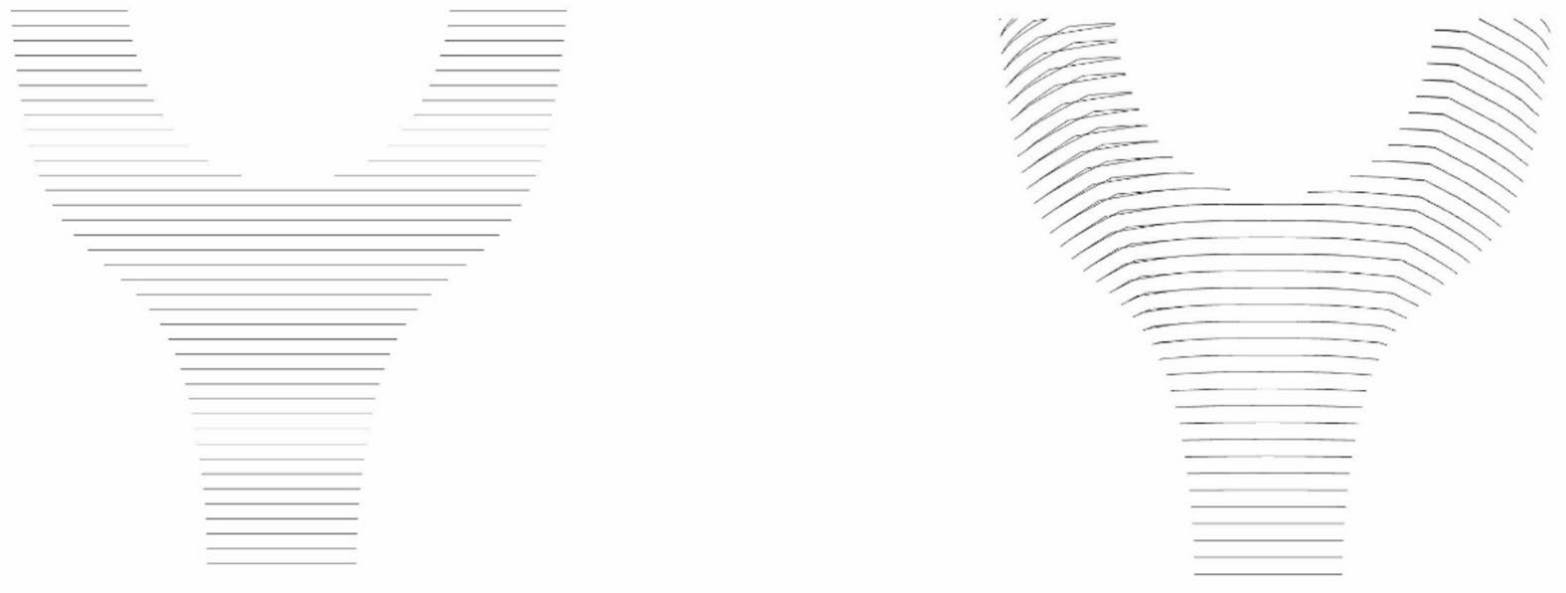
Slicing methods: planar in z-direction (left) and equidistantly (right).

Regarding the slicing of the main body, two principal approaches can be employed: planar slicing in the z-direction and the equidistant slicing method (Fig. 4).

**Fig. 5:**
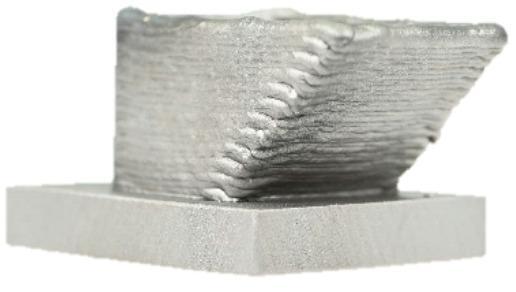
Example of planar slicing with additionally added layer in the overhang (23 layers for 0° overhang, 32 layers for 45° overhang).

The key distinction between these methodologies lies in the fact that the equidistant slicing method incorporates a consideration for the increased layer density required in overhang areas. The necessity for additional layers in overhang regions is exemplified in Fig. 5. For the 45° overhang side, 32 layers were needed to achieve an equivalent total height, compared to 23 layers at the vertical side when using the same DED-Arc process parameters during printing. This difference arises because the effective vertical build height per layer is reduced on the inclined surface by $\:\mathrm{cos}(45^\circ\:)\approx\:0.7$. An adjustment to the printing parameters would affect cooling behavior and thus the resulting surface topography and mechanical properties in the overhang area and is therefore not considered.

To avoid the manual addition of these supplementary layers, the equidistant slicing method was implemented in the fabrication process of the Y-Node. This methodological choice ensures a more adaptive and efficient distribution of layers, particularly in regions with overhang, thereby optimizing the overall structural integrity of the manufactured component by reducing start/stop-points.

#### Path planning

Following the slicing procedure, the transformation of layers into a robot path becomes imperative. In this phase, each path is endowed with manufacturing parameters, including but not limited to travel speed, process specifications, the positioning of the tilt-turn table, and torch orientation. Emphasis is placed on the critical aspects of torch orientation and the coordinated movement of the tilt-turn table. Factors such as collision avoidance, accessibility, and welding position demand meticulous examination and consideration within this stage.

**Fig. 6:**
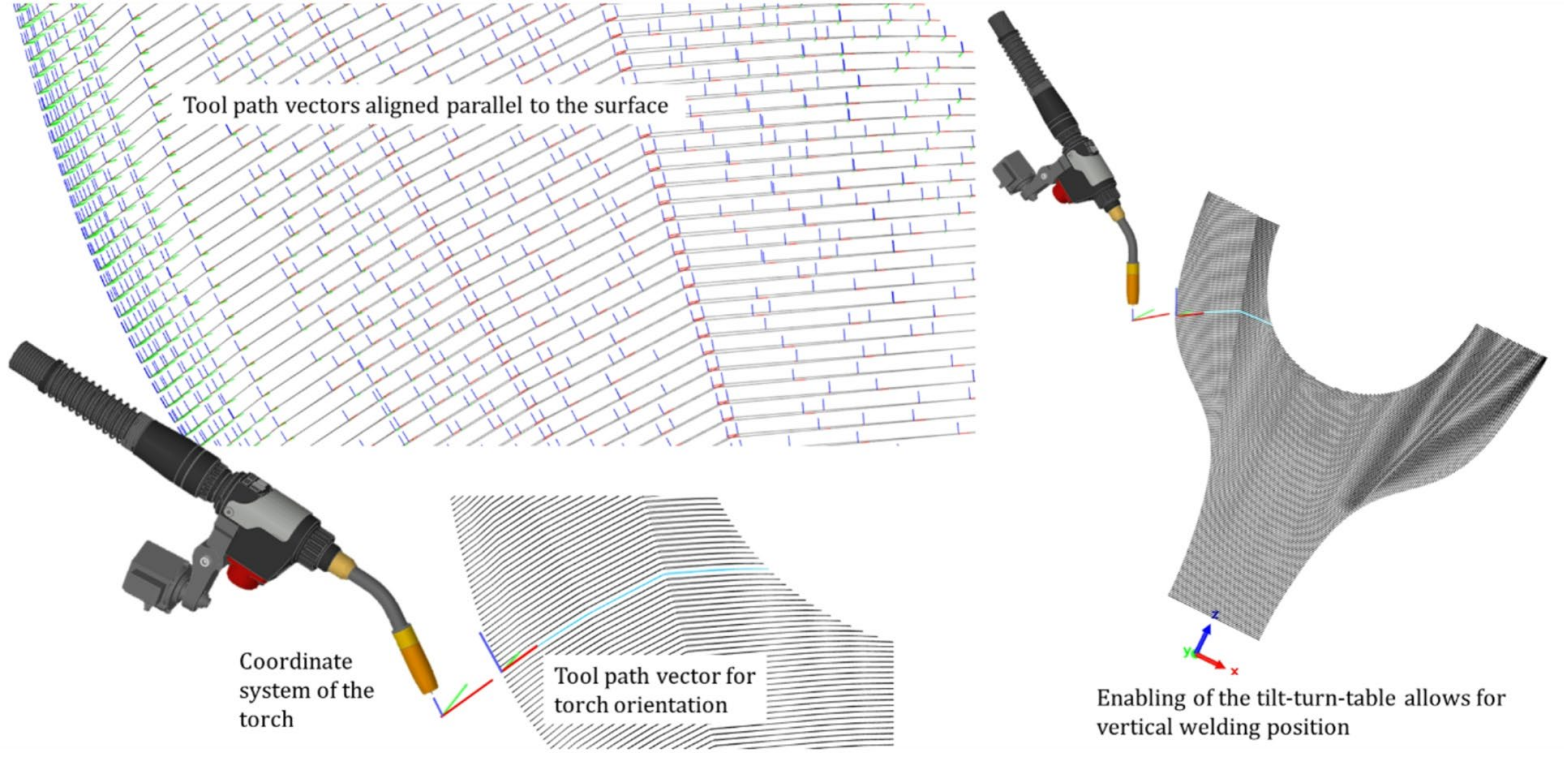
Alignment of vectors for torch orientation (blue) tangential to the surface (left) and activation of the tilt-turn table to allow for vertical printing position in overhangs (right).

Achieving precise deposition of successive layers is facilitated by aligning the vectors for torch orientation tangentially to the surface, see Fig. 6. This alignment ensures meticulous layer-by-layer deposition, enabling the realization of higher degrees of overhang. Notably, adopting a vertical printing position by using the tilt-turn table proves advantageous for optimizing surface quality. In this position, the molten pool is shielded from the force of gravity, being supported by the underlying layer, thus contributing to enhanced surface finish.

The fabrication of the second component of the Y-Node, namely the bridge, involved an adjustment in the building direction. A nuanced manufacturing approach was applied to this section. The layers constituting the bridge were deposited from each side of the main body in an alternating way, careful to avoid collision between the torch and the already built component. Consequently, the gap between the two sides progressively diminished until the bridge reached closure in the middle. In this context, the vectors were initially aligned tangentially to the surface, mirroring the approach employed for the main body. However, as the two sides of the bridge approached each other, these vectors assumed a more vertical orientation to accommodate the converging structure effectively. This approach ensures precise layer deposition as the bridge sections merge to complete the Y-Node.

### Manufacturing of the demonstrator

**Fig. 7:**
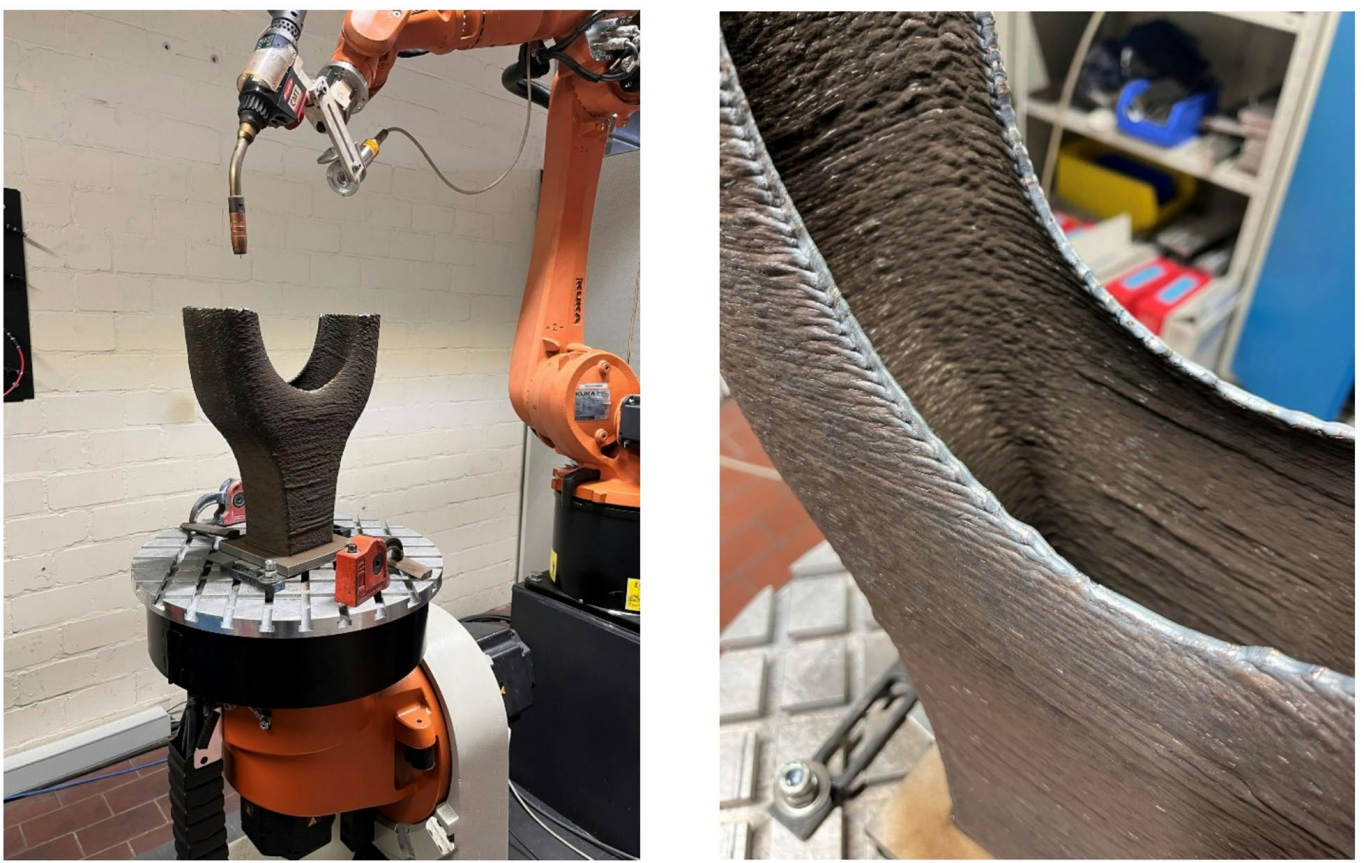
Finalization of the main body of the Y-Node (left) and close-up of the TIG-smoothened contour (right).

Upon completion of the design, slicing and path planning phases, the manufacturing of the Y-Node commenced with the preparation and clamping of a 160 × 160 mm base plate onto the tilt-turn table. The contact tip-to-workpiece distance was set at 15 mm. By progressing layer by layer, the main body of the node took shape. However, in the region where the rectangular cross-section transitions into two round cross-sections, an intentional open path was employed, see Fig. 7. This design restriction resulted in start/stop-points being situated on the periphery of the main body, yielding a relatively irregular and undulating edge.

To smoothen this surface irregularity and facilitate a seamless junction between the main body and the bridge, Tungsten Inert Gas (TIG) welding was applied to refine the edges. This intervention allowed the creation of a remarkably smooth and uniform edge, ensuring optimal conditions for the subsequent application of the first layer of the bridge without risking lack of fusion.

Upon adding the bridge, it became apparent that the main body exhibited minor distortions attributable to suboptimal clamping. Consequently, a slight misalignment between the two components (main body and bridge) of the node ensued. The distortion of the base plate can significantly impact the final structure due to the increasing misalignment throughout the manufacturing process, which can be accounted for by utilizing a thicker base plate. Even a minor distortion in the base plate can result in deviations that amplify as the layers are successively deposited. The distortion of the base plate disrupts the intended planar orientation for subsequent layers. As the layers build upon each other, any misalignment introduced at the base becomes magnified, especially when constructing complicated or vertically aligned features. This cumulative effect can lead to deviations on the top of the produced structure, as each layer is influenced by the misalignment of the preceding ones.

**Fig. 8:**
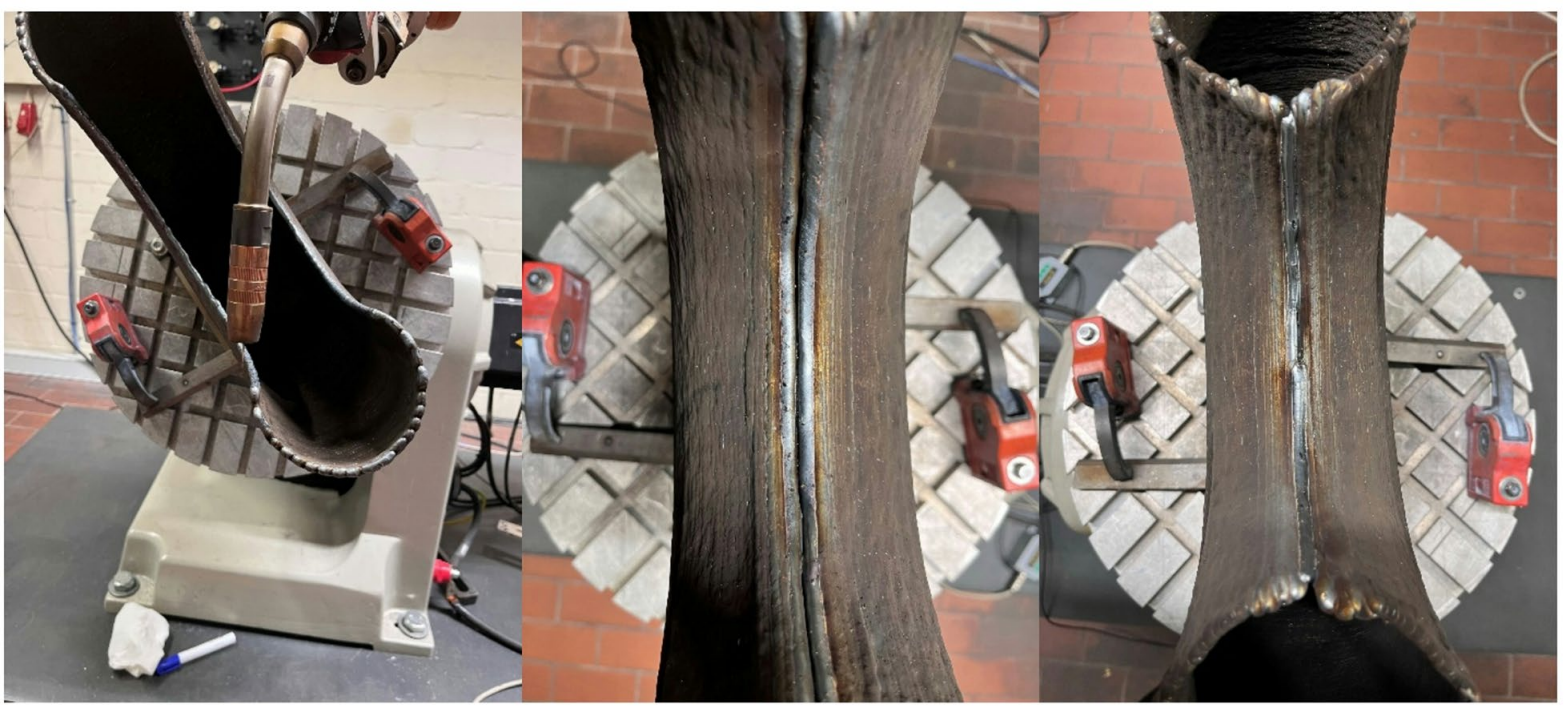
Manufacturing of the bridge for closing the Y-Node.

The culmination of challenges materialized during the final weld bead application for closing the bridge, see Fig. 8. The distortion necessitated the division of the last weld bead to ensure complete penetration of both sides of the bridge, representing a strategic adaptation to overcome the encountered misalignment and achieve a structurally sound final configuration.

It is noteworthy that a manual adjustment of robot paths became necessary in the bridge region. The decision to manually shift the paths was prompted by the recognition that connecting the last layers at an 180° angle posed inherent challenges. Specifically, concerns regarding potential collisions between the torch and the workpiece surface were identified. Additionally, achieving satisfactory deposition of weld beads within the constrained geometry of the bridge area proved to be a challenging aspect of the manufacturing process.

It is important to acknowledge that while these challenges necessitated manual intervention and adjustments, they provided valuable insights into the complexity of design, slicing and manufacturing larger structures. The attention to detail and adaptability in response to challenges underscore the iterative nature of additive manufacturing processes. By addressing these challenges thoughtfully, valuable lessons are gained, contributing to the refinement and optimization of future large-scale DED-Arc manufacturing projects.

## Digital representation of a Y-Node

In the construction sector, large components are frequently unique and not intended for serial production, thus rendering a verification process based on prototyping and coupon testing infeasible. The present study proposes a structural design process based on the prediction of component performance and quality management. The manufacturing process can be monitored, and predictions on the resulting part properties can be drawn based on the obtained data. In the following, the concept of a Digital Twin (DT) for DED-Arc steel components is described, and applied to the printed Y-Node. A prediction of the stress distribution of the component based on the DT data was performed, using data that had previously been captured.

### Digital linkage of design, manufacturing and geometry data

**Fig. 9:**
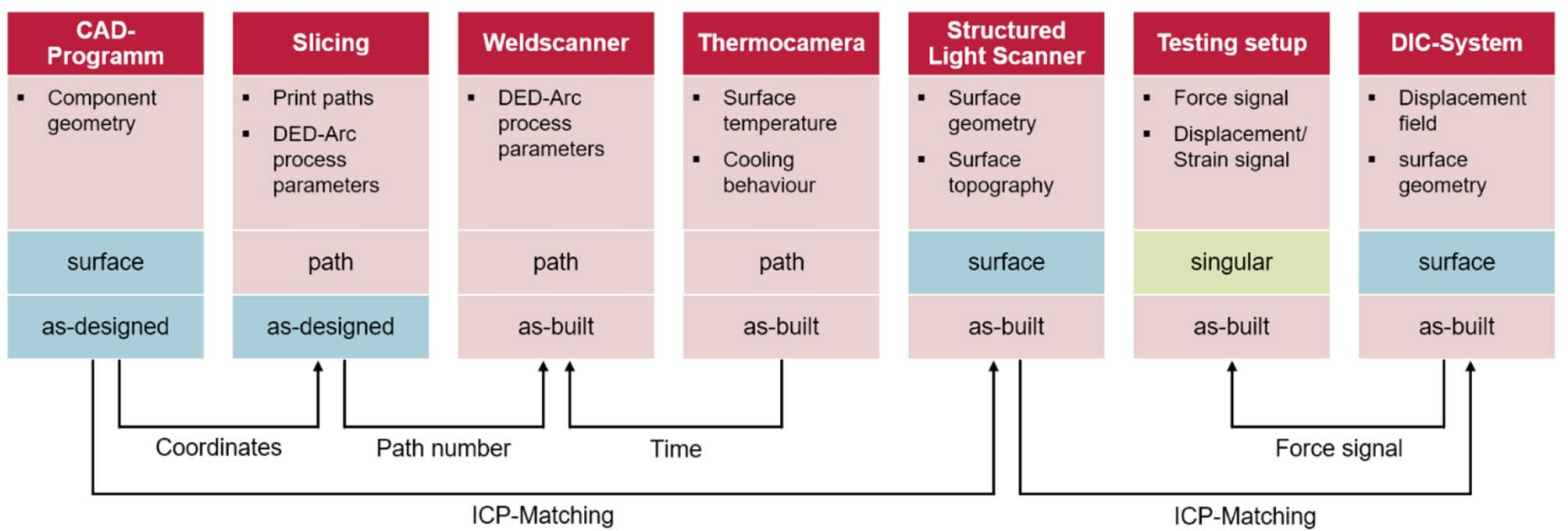
Diagram of the proposed DT data registration workflow.

It is evident that the manufacturing process influences the design, manufacturing strategy and component performance. Consequently, localized information is required to comprehend the correlation of as-designed geometry, print strategy, process data and as-built geometry (see Fig. 9). The data generated and gathered during the planning, production and testing stages is heterogeneous, with the resulting data structure comprising point clouds, paths, surfaces and volumes. Given the potential for data originating from disparate sources, a transformation process is necessary to align these different datasets with a common spatial and temporal framework for context awareness^25^. This transformation and alignment step forms part of the communication and data-exchange layer that connects the physical process, process monitoring and inspection data^26^. The process necessitates meticulous planning and care during data acquisition to ensure accurate parameter determination. The feasibility of merging datasets is contingent upon the availability of identifiable reference markers within the respective datasets^27^.

Three methods are implemented in the DT to link different types of data. Surfaces are linked using an Iterative Closest Point (ICP) algorithm^28^. The initial design geometry (as-designed) serves as the reference system. All other surface data is mapped back to the coordinate system of the as-designed geometry. For the linking of path related data, each path is assigned a unique identifier and a projected length. Slicing the component prior to manufacturing results in the transformation of volume geometry to path related data. This coordinate data transformation is the basis for the back-transformation of path-related process data to the surface. Time signal or trigger signals are used for temporal context during the printing for capturing the DED-Arc process parameters and the resulting temperature fields as well as to link singular data, such as the force signal during testing.

A particular challenge when comparing geometry data from different timesteps or data sources along the manufacturing process is distortion, e.g. due to different cooling behavior. No compensation has been made for this effect, however, given the components’ relatively small size, the effects of distortion are assumed to be negligible in terms of spatial context. Nevertheless, as component size increases, this influence becomes increasingly relevant. One method of quantifying this influence could be online geometry acquisition during the manufacturing process with fixed reference points on the base plate.

### Comparison of as-designed and as-built geometry

**Fig. 10:**
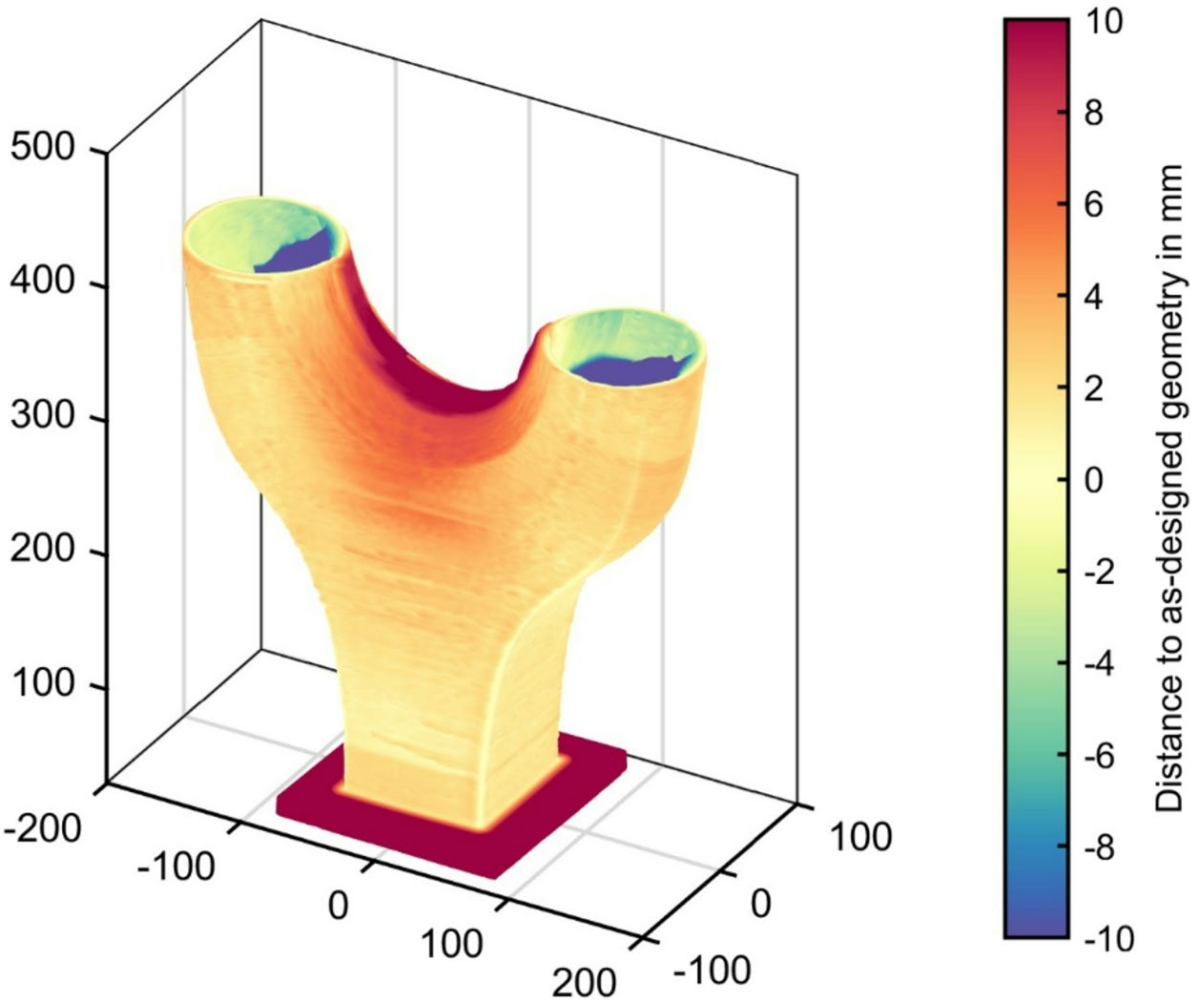
Comparison of the as-planned geometry (CAD-file) with the as-built geometry.

To evaluate the distortion and geometrical accuracy of the build node, a comprehensive structured light 3D scan of the as-built component was performed and compared to the as-designed geometry, see Fig. 10. The scan was performed with an Artec Eva-S device. Based on the data of the digital representation of the Y-Node, the best-fit distance of the as-designed center surface to the 3D scan of the as-built surface is shown. The analysis revealed that deviations arising from the slight distortion of the base plate fell within a range of ± 5 mm. Especially for large components, even minimal distortion of the base plate can have a significantly amplified effect with increasing distance to the baseplate. Notably, the most prominent variations were observed in the section requiring closure of the bridge. The large deviation in the blue area results from the post-processing of the 3D scan data to obtain a closed volume model.

### Assessing the manufacturing data

**Fig. 11:**
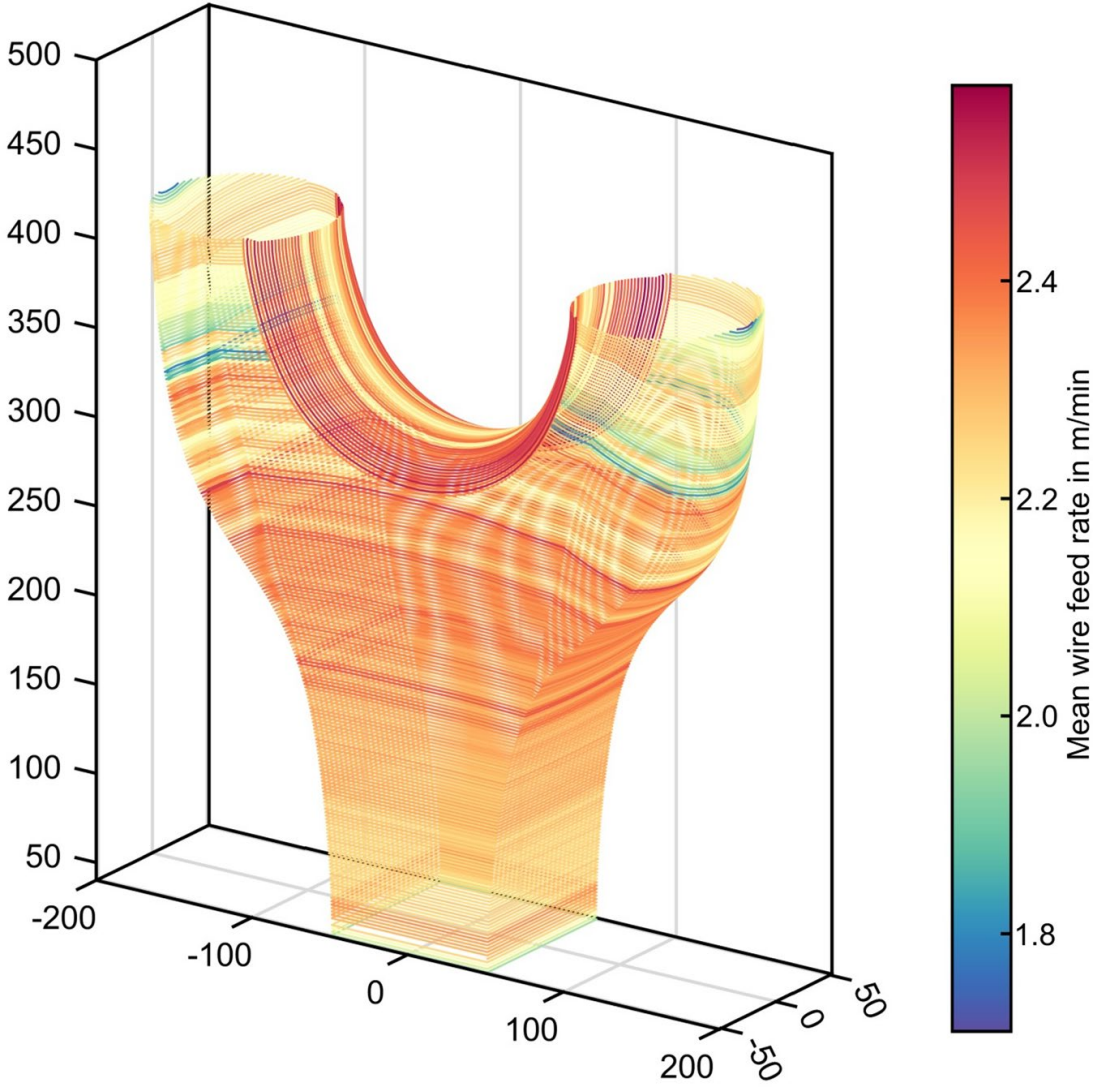
Visualization of mean wire feed rate per layer during manufacturing.

To visualize the DED-Arc process data collection in the DT framework, the mean wire feed rate per layer in m/min is shown in Fig. 11. Along the build height in the central body of the Y-Node there is a gradual shift towards higher wire feed rates, whereas in the arm regions the wire feed rate is decreased again. During the deposition of the material in the central body, slight variations in wire feed rate can be observed, resulting from the internal control of the power source, which adapts current and thus wire feed rate in order to maintain a stable arc when e.g. the contact tip to workpiece distance varies or when the contact tube is either dirty or worn. In the process data of the bridge region between the arms, the change in path orientation of the deposition, as described in the section "Slicing and path planning", is visible. Additionally, it can be seen that for the first layers, the internal control led to comparatively high wire feed rates of up to 2.4 m/min which are reduced towards the center of the bridge to the initial value of 2.0 m/min.

Although the influence of the main DED-Arc process parameters on the mechanical strength has been investigated, manufacturing parameters beyond direct welding affect the resulting material behavior and surface topology, including print overhang angle, start point location, print direction, and print pauses^20^. To determine the influence of the printing strategy on the stress response of the Y-Node, the slicing commands were assessed.

The overhang angles can be derived from the geometry and robot kinematics. For the Y-Node, a tilt-turn table was used, so the rotation of these external axes must be considered. It is necessary to calculate the position of the part at each specific time step in the manufacturing process and to calculate the build direction relative to the direction of gravity, and therefore the overhang angle and the tool angle.

**Fig. 12:**
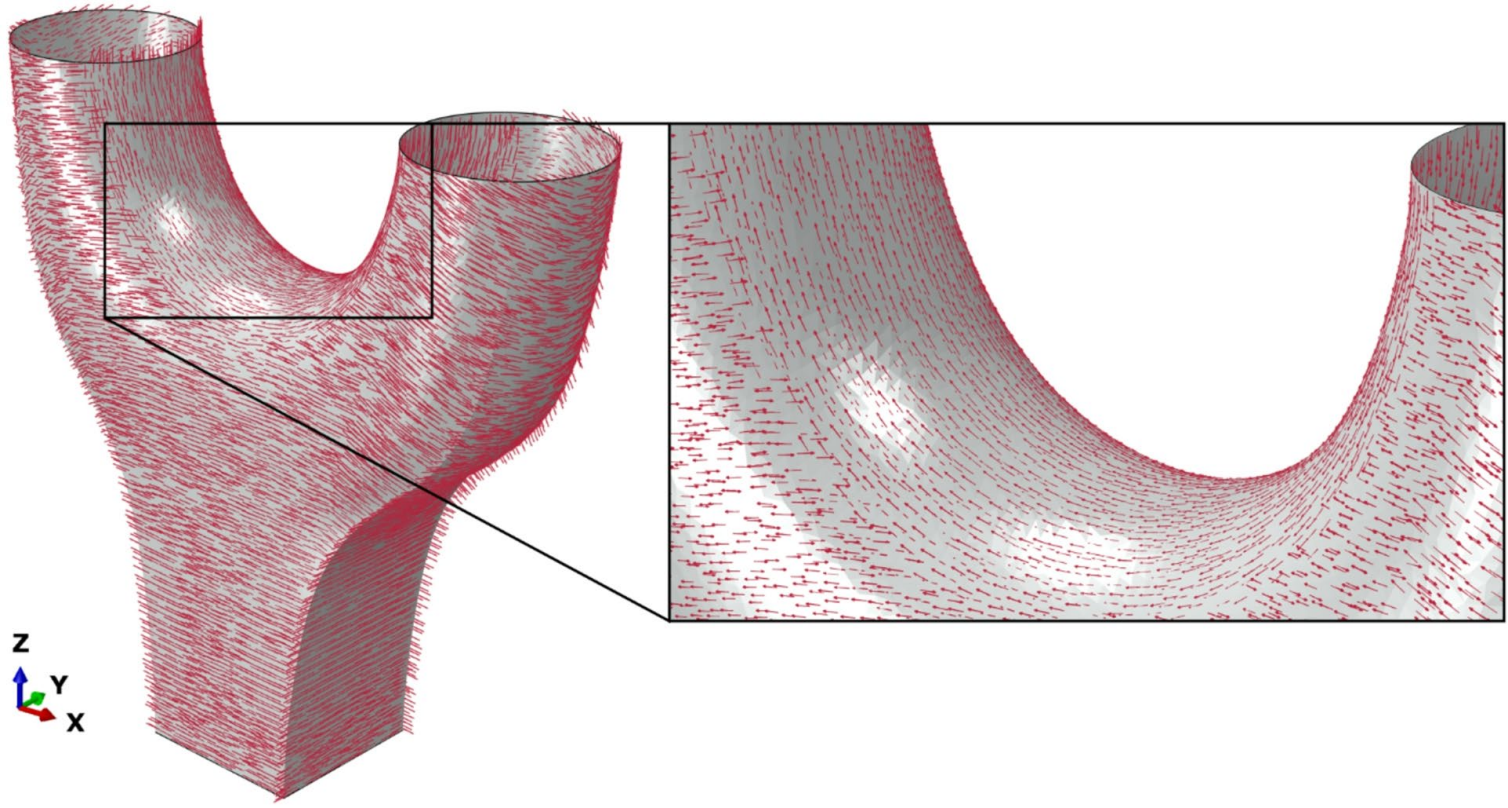
Global captured path related print direction (left), zoom into the print direction at the bridge area (right).

Figure 12 describes the assessment of the calculated layer orientation relative to the print path. The manufacturing strategy described in the section "Slicing and path planning" can be seen in the printing orientation. The interface is clearly visible in the area of the arms. The data also indicate that, from a material perspective, no overhang is present in the area of the arms (see Fig. 12 (right)), as the tilt-turn table was positioned during printing to ensure that the layer was deposited in a manufacturing position close to 0 degrees. Otherwise, an overhang would have a local influence on the loading^20^.

The availability of these results enables process data to offer important insights into component behavior. The linking of geometry and process data is therefore an important requirement for future methods for predicting component performance based on DT data^27^.

### Prediction of component performance

The as-designed geometry in combination with the captured path-related process data was used for a numerical stress analysis of the component performance undergoing vertical loading. A geometrically and materially nonlinear analysis with imperfections (GMNIA) was performed using Abaqus CAE 2017 and 3-node triangular general-purpose shell elements with finite membrane strain formulations. Based on an anisotropic material model for the HSLA steel^9^, the directional data was used for the alignment of the normal direction the elements. The mesh is based on the as-designed geometry, which was re-meshed using an isotropic explicit remeshing technique that regularizes the size and aspect ratio of the elements. To limit the number of different material orientations in the model, similar orientations were bundled by comparing the Euler transformation angles of each material orientation to a fixed number of prescribed orientations, specified by 5° angles between each other, and selecting the closest match. The load was applied via the two arms in z-direction. The displacements were fixed at the base point.

**Fig. 13:**
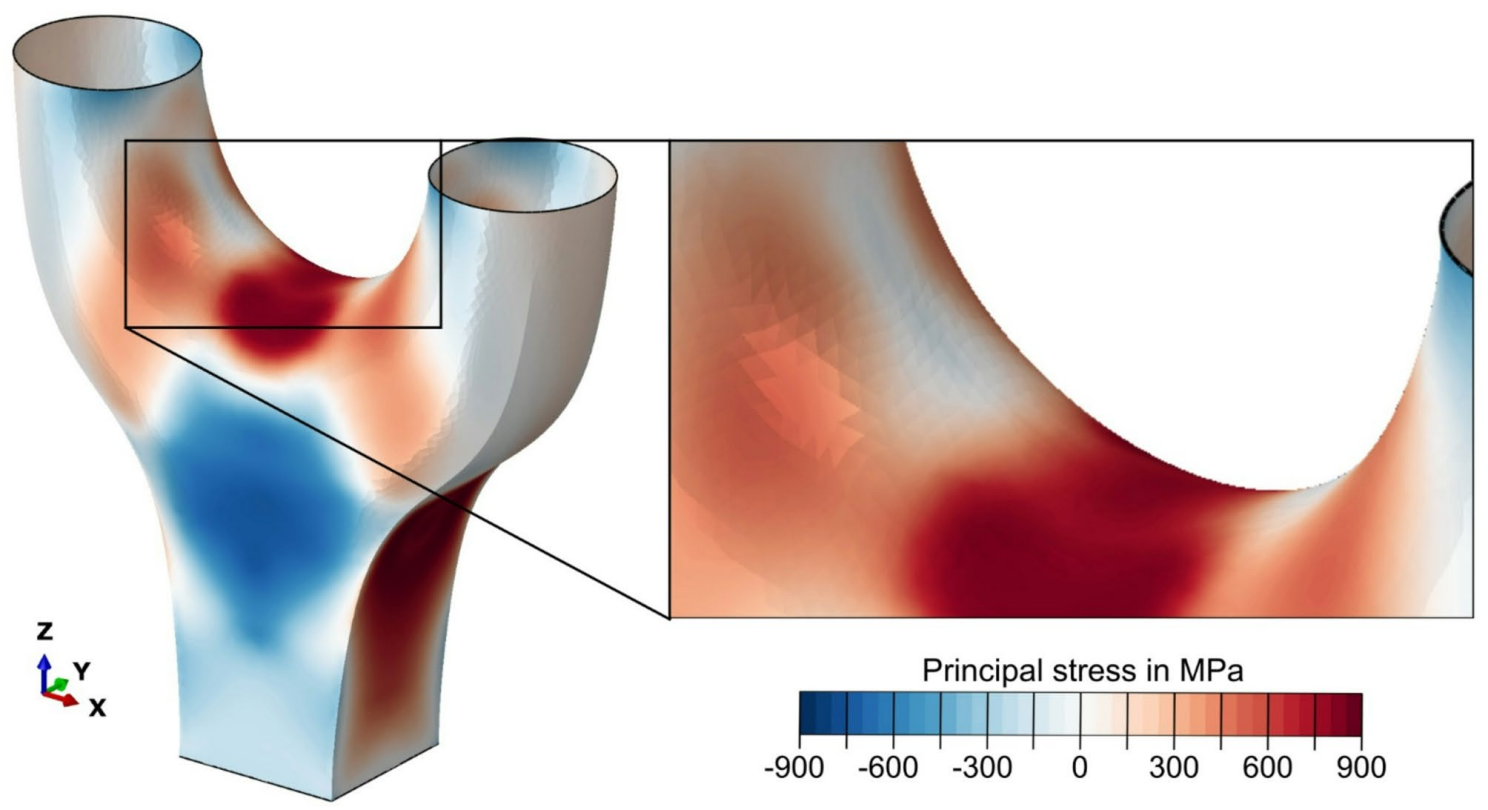
Global simulated maximum principal stress distribution based on process and robot data (left), zoom into stress distribution at the bridge area (right).

Figure 13 shows the maximum principal stress of the component based on the GMNIA at the yield stress, which was derived from uniaxial quasi-static tensile tests^9^. A distinct stress concentration can be seen between the two arms. This concentration can be expected as a global response with the chosen load and geometry. The influence of the manufacturing direction on the stress distribution can be seen in the arm and in the area of the bridge between the arms, where the printing direction has been adjusted (see Fig. 12 (left)) to account for the anisotropic material behavior^20^. Also, there are major stress concentrations at the connecting points of the two arms.

## Summary

This report shows the design, manufacturing and evaluation of a DED-Arc Y-Node for the construction industry, explicitly targeting the challenges during each step. Although the geometry of the Y-Node was chosen arbitrarily as a connection between a rectangular and two circular hollow sections, the challenges of slicing, the manufacturing strategy and the performance prediction are highlighted in detail. It is shown that geometric freedom goes along with an increase in complexity in the path planning and manufacturing strategy. It is therefore necessary to consider the constraints of the manufacturing process at an early stage of the design process. For the future, the slicing process should account for needs of manufacturability and loading conditions already in initial stages of process planning. For the link between design strategy, manufacturing process, as-designed and as-built geometry data a framework for a DT structure is proposed and established. The temporal and spatial linking of surface, path and singular data in one coordinate system is described in detail. It is emphasized that the linking of data must already be considered before and during data acquisition to account for distortions during manufacturing. The DT data is a fundamental prerequisite for the development of methods for predicting component behavior and was used to predict the stress distribution for a defined load case. The results of this work underline that a full workflow from designing and manufacturing in a structured digital representation is essential for large-scale DED-Arc printing.

The incorporation of online geometry scans and adaptive path planning during the manufacturing of large-scale components is pivotal for maintaining precision and mitigating deviations of the as-built from the as-designed geometry. The integration of 3D laser or structured light scanning systems after each layer deposition facilitates real-time assessment, enabling prompt identification of geometric variations. Leveraging 3D scanning technology provides a robust means of comparing the fabricated structure against the intended design, allowing for early detection and correction of deviations.

Moving forward, a strategic focus on adaptive path planning is crucial for iterative adjustments based on continuous geometry scans. This approach ensures that the manufacturing system dynamically responds to evolving geometric conditions, maintaining alignment with the design intent throughout the additive manufacturing process.

For future large-scale component manufacturing, the implementation of closed-loop feedback systems stands out as a promising approach. The automated corrections of process parameters and deposition paths derived from real-time monitoring data offer a proactive means to minimize the risk of cumulative deviations, enhancing overall accuracy and reliability in the fabrication of complex and intricate structures. By embracing these advanced strategies, manufacturers can significantly improve the quality and consistency of large-scale components produced through additive manufacturing.

## Data Availability

The data can be accessed under https://doi.org/10.5281/zenodo.17192191.
